# Interpretation, Confounding, and Context in Dementia Care for Older Adults Living Alone: Letter to the Editor

**DOI:** 10.31662/jmaj.2026-0055

**Published:** 2026-03-27

**Authors:** Yudai Kaneda

**Affiliations:** 1 Clinical Training Center, Jyoban Hospital of Tokiwa Foundation, Fukushima, Japan

**Keywords:** living alone, dementia care, physical function, confounding bias, Japan

I read with interest the recent article by Maeshima et al. ^(1)^ on dementia care challenges among older adults living alone in Japan. I commend the authors for addressing this important issue in a real-world clinical setting. I offer several comments for clarification and further discussion.

First, I noted an inconsistency in the reporting of physical function. In Table 1, both grip strength (right: 18 vs. 21 kg, p = 0.0139; left: 14 vs. 19.3 kg, p < 0.0001) and the Rivermead Mobility Index (RMI: 13 vs. 14, p = 0.0014) were lower in the group of older adults living alone, yet the Results and Discussion sections describe these values as significantly higher. Since both grip strength and the RMI are standardized measures in which higher values indicate better physical function, resolving this discrepancy is essential, as the authors’ interpretation hinges on the assumption that older adults living alone maintain superior physical capabilities despite cognitive decline. Confirming which data are accurate would enhance the transparency and clinical relevance of the findings.

Second, I am concerned about the comparability of the groups. The group of older adults living alone was significantly older (median 83 vs. 80 years, p = 0.0022), more likely to be female (77.2% vs. 49.8%, p = 0.0001), and had slightly fewer years of education (median 11 vs. 12, p = 0.0435). All these variables are known to affect both cognitive and physical performance ^(2)^. Since only unadjusted comparisons using Mann-Whitney U and χ^2^ tests were performed, residual confounding cannot be ruled out. I encourage the authors to consider multivariable regression or propensity score methods to account for these differences, as such approaches are standard in geriatric and dementia research when analyzing observational data ^(3)^.

Finally, while the location of the memory clinic is not explicitly stated, the authors’ affiliations and IRB approval indicate that the study was conducted in Obu City, Aichi Prefecture, Japan―a car-dependent suburban area. A local survey of Obu residents aged ≥65 years reported that 75.5% were active drivers, and 24.5% were non-drivers ^(4)^. As frail older adults often rely on others’ vehicles for medical visits ^(5)^, those living alone who reached the memory clinic may represent a select subgroup with preserved mobility or family support. This sampling feature may limit the generalizability of the findings to a population with more transportation disadvantages. Making the clinic’s location explicit would help contextualize the results and better assess their geographic applicability.

I thank the authors for their valuable work and hope these comments contribute to further clarification and refinement of this important area of research.

## Article Information

### Author Contributions

Conception and writing - original draft: Yudai Kaneda

### Conflicts of Interest

None
